# Epidemiological Differences in Hajj-Acquired Airborne Infections in Pilgrims Arriving from Low and Middle-Income versus High-Income Countries: A Systematised Review

**DOI:** 10.3390/tropicalmed8080418

**Published:** 2023-08-17

**Authors:** Hashim A. Mahdi, Mohammed Alluhidan, Abdulrahman B. Almohammed, Mohammad Alfelali, Ramon Z. Shaban, Robert Booy, Harunor Rashid

**Affiliations:** 1 National Centre for Immunisation Research and Surveillance, The Children’s Hospital at Westmead, Westmead, NSW 2145, Australia; 2 The Children’s Hospital at Westmead Clinical School, Faculty of Medicine and Health, The University of Sydney, Westmead, NSW 2145, Australia; 3 Department of Public Health, College of Health Sciences, Saudi Electronic University, Jeddah 23442, Saudi Arabia; 4 General Directorate for National Health Economics and Policy, Saudi Health Council, Riyadh 13315, Saudi Arabia; 5 College of Medicine, Imam Muhammad Ibn Saud Islamic University, Riyadh 11564, Saudi Arabia; 6 Family and Community Medicine Department, Faculty of Medicine in Rabigh, King Abdulaziz University, Jeddah 25732, Saudi Arabia; 7 New South Wales Biocontainment Centre, New South Wales Ministry of Health, Westmead, NSW 2151, Australia; 8 Faculty of Medicine and Health Susan Wakil School of Nursing, The University of Sydney, Sydney, NSW 2006, Australia; 9 Public Health Unit, Centre for Population Health, Western Sydney Local Health District, North Parramatta, NSW 2151, Australia; 10 Sydney Infectious Diseases Institute, The University of Sydney, Westmead, NSW 2145, Australia

**Keywords:** airborne infections, Hajj, high-income countries, low and middle-income countries, pilgrims

## Abstract

This systematised review aims to compare the epidemiological patterns of Hajj-acquired airborne infections among pilgrims from low and middle-income countries (LMIC) versus those from high-income countries (HIC). A PubMed search was carried out for all published articles before February 2023, using a combination of MeSH terms and text words. The Newcastle–Ottawa Scale (NOS) was used to assess data quality. From a total of 453 titles identified, 58 studies were included in the review (LMIC = 32, and HIC = 26). In the pooled sample, there were 27,799 pilgrims aged 2 days to 105 years (male: female = 1.3:1) from LMIC and 70,865 pilgrims aged 2 months to 95 years (male: female = 1:1) from HIC. Pilgrims from both HIC and LMIC had viral and bacterial infections, but pilgrims from HIC tended to have higher attack rates of viral infections than their LMIC counterparts. However, the attack rates of bacterial infections were variable: for instance, pilgrims from LMIC seemed to have higher rates of meningococcal infections (0.015–82% in LMIC vs. 0.002–40% in HIC) based on the study population, but not *Mycobacterium tuberculosis* (0.7–20.3% in LMIC vs. 38% in HIC). Targeted measures are needed to prevent the spread of airborne infections at Hajj.

## 1. Introduction

Hajj is an annual religious mass gathering (MG) held in Makkah, the Kingdom of Saudi Arabia (KSA), which, during a non-pandemic year, attracts more than two million pilgrims to attend the pilgrimage [1]. During the Hajj pilgrimage, the climatic conditions and inevitable overcrowding of pilgrims in Makkah and the surrounding holy sites significantly increase the likelihood of contracting and transmitting infectious diseases [2]. Pilgrims come from over 180 countries, including both high-income and low-income nations, with various unique cultural, nutritional and health behaviour backgrounds. Pilgrims originating from regions with variable disease profiles present one of the most significant challenges to the host country. Certain diseases or infectious agents may be prevalent in some regions but not in others [3], which makes it difficult for the host country to apply a uniform preventive guideline. Additionally, pilgrims from countries with a low burden of disease may acquire infection from pilgrims from countries with a high burden of disease, due to their close contact in confined spaces in accommodation and during rituals [2]. Also, many pilgrims, particularly those from developing countries, may have limited access to adequate healthcare services and preventative measures, e.g., vaccinations before they commence their journey [4]. Such diversity in the pilgrim population might determine the burden of health risks acquired during this MG event. 

In particular, airborne infections are a major public health issue and a significant cause of morbidity at Hajj. Pneumonia is the leading cause of hospitalisation during Hajj and the second or third most common cause of admission to the intensive care unit (ICU) during Hajj [5]. Furthermore, many airborne infections have emerged in recent decades, including severe acute respiratory syndrome (SARS) in 2003, the influenza A (H1N1)pdm09, the Middle East respiratory syndrome coronavirus (MERS-CoV) in 2012, and, more recently, the novel coronavirus disease 2019 (COVID-19) in 2019, presenting a major public health and infection control challenge for both the authorities in KSA and the national authorities in the pilgrims’ countries of origin [6,7].

A handful of systematic reviews have provided a comprehensive overview of available evidence on the burden of respiratory infections in Hajj [5,8,9,10,11]; however, no study was undertaken to compare the burden of infections at Hajj among participants from low and middle-income countries (LMIC) and high-income countries (HIC). This comparison is important, as it can inform future public health policy and identify potential prevention and control strategies. Therefore, this systematised review aims to compare the epidemiological patterns of Hajj-acquired airborne infections among pilgrims from LMIC versus HIC. By the term ‘airborne infection’, we mean any infection transmitted by respiratory route, irrespective of whether it primarily causes respiratory infection or not, so infections like ‘meningococcal disease’ were also included.

## 2. Materials and Methods

### 2.1. Search Strategy and Study Selection

This review was conducted and reported following the Preferred Reporting Items for Systematic Reviews and Meta-Analyses (PRISMA) guidelines [12]. However, we did not attempt to register this systematised review with PROSPERO or other registry databases as the process takes much longer during the COVID-19/post-COVID-19 period, and we did not intend to delay this important systematic review.

The PubMed electronic database was comprehensively searched for all relevant articles published from inception to February 2023. Also, a manual search was conducted to identify additional potentially eligible articles by reviewing the reference lists of the included studies. The search strategy was developed using a combination of MeSH terms and keywords, including: ‘Hadj’ OR ‘Hadz’ OR ‘Hajj’ OR ‘Mecca’ OR ‘Makkah’ OR ‘pilgrimage’ OR ‘pilgrims’ AND ‘respiratory tract infection’ OR ‘airborne infection’ OR ‘acute respiratory infection’.

Three independent reviewers (HAM, M. Alluhidan, and HR) evaluated the retrieved studies for eligibility by screening titles and abstracts, selected relevant manuscripts based on the inclusion criteria, and then reviewed the full texts of each paper potentially meeting the inclusion criteria. If any discrepancies or disagreements arose, they were resolved through discussion among the researchers. The inclusion criteria included primary studies reporting microbiological data on viral or bacterial respiratory infections involving Hajj pilgrims of any age, gender, or country of origin that employed a laboratory-based diagnostic method. Only English language articles were considered for this review. Studies conducted outside the Hajj setting or involving non-pilgrim participants rather than Hajj pilgrims or reporting only clinical or self-reported respiratory symptoms or infections without laboratory-confirmed data were excluded. The pilgrims’ countries of origin were classified based on the 2021–2022 World Bank Classification for countries by income (https://datatopics.worldbank.org/world-development-indicators/the-world-by-income-and-region.html accessed on 2 March 2023).

### 2.2. Data Extraction and Synthesis

The data were independently extracted from each included article by two authors (HAM and M. Alluhidan) into an extraction spreadsheet. The extracted data were then cross-checked for accuracy by another reviewer (HR). The extracted data included the year of study, selection method/case ascertainment, testing methods, study sample, nationality, age and gender of participants, risk factors, influenza and pneumococcal vaccine uptake, and burden of airborne infections. 

The studies were heterogeneous in terms of study sites, diagnostic tests applied, and research outcomes assessed; hence, a meta-analysis or correlation analysis has not been attempted. However, a narrative synthesis was carried out by providing the upper and lower ranges of proportions for comparative variables and attack rates of airborne infections.

The study quality was assessed by two authors (HAM and HR) using the Newcastle–Ottawa Scale (NOS), in which an observational study is scored based on a ‘star system’ across three broad domains: the selection of the study groups; the comparability of the groups; and the ascertainment of either the exposure or outcome of interest. Conventionally, a score of 6–9 is considered good quality, a score of 3–5 as average and a score of 0–2 as poor (http://www.ohri.ca/programs/clinical_epidemiology/oxford.asp accessed on 5 April 2023).

## 3. Results

### 3.1. General Description of Included Studies and Quality Assessment

**Figure 1:**
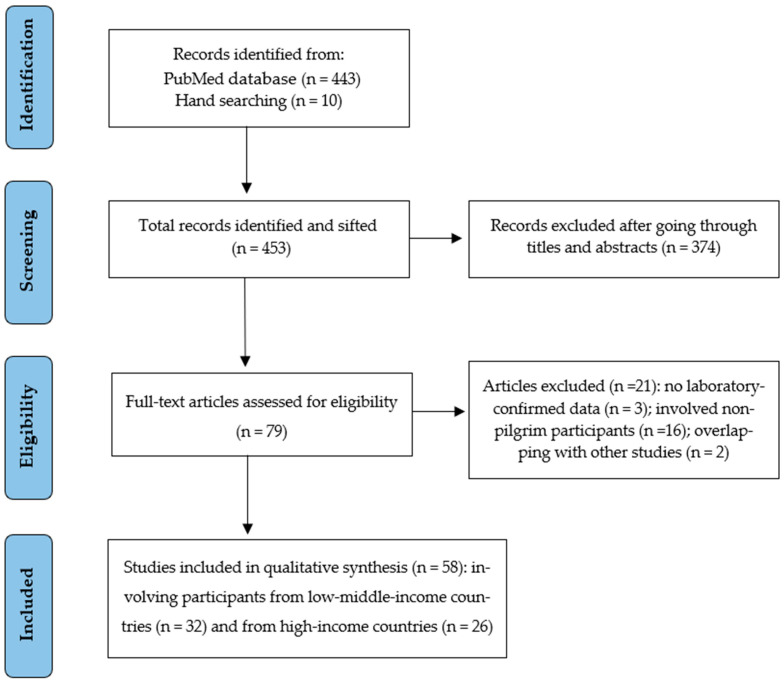
PRISMA flowchart of the search strategy.

As shown in the PRISMA flowchart (Figure 1) database searches and hand searching resulted in a total of 453 hits; these 79 full texts were reviewed, of which 58 studies finally met the inclusion criteria: 32 involved pilgrims from LMIC (Table 1) and 26 from HIC (Table 2). The studies involved pilgrims who attended Hajj from 1987 to 2021, with study sample sizes varying widely from 7 to 58,428 participants. The studies were heterogeneous in terms of study sites, diagnostic tests applied to identify the causative agents of infections (such as polymerase chain reaction (PCR) or viral/bacterial culture), and outcomes assessed. 

Pilgrims were recruited from a variety of settings, including healthcare facilities in Makkah and/or Al Madinah during Hajj [13,14,15,16,17,18,19,20,21,22,23,24,25,26,27,28,29,30], tents in Mina [31,32,33,34,35], entry or exit points in Saudi Arabia [36,37,38,39,40], arrival or departure lounges/waiting rooms of pilgrims’ home country airports [41,42,43,44,45,46,47,48,49,50,51,52,53,54], hospitals in pilgrims’ home countries immediately after their return [55,56,57,58,59], or travel clinics before their departure from home countries [60,61,62,63,64]. The burden of viral respiratory infections alone was reported by 28 studies [14,18,20,22,28,29,31,33,34,36,37,41,42,43,44,45,46,47,51,52,53,55,57,58,61,65,66,67]; bacterial respiratory infections alone by 22 studies [13,15,16,17,19,23,24,27,30,32,35,40,48,49,50,54,56,63,64,68,69]; and both viral and bacterial airborne infections by 8 studies [21,26,38,39,59,60,62,70].

**Table 1:** Summary of included studies involving participants from low and middle-income countries.

Author, Publication Year	Study Year	Participants’ Nationalities (%)	Selection Method/Case Ascertainment	Testing Method	Sample Size	Mean/Median Age (Range)	Male: Female	Risk Factors (%)	Vaccine Uptake (%)	Detected Diseases Name	Disease Burden n (%)
AlBarrak et al., 2018 [13]	2016	Majority from LMIC: Indonesian (22.3) Egyptian (10.2) Indian (9.8)	Adults with radiologically confirmed pneumonia admitted in general hospitals in Makkah and Al Madinah	Urine antigen test was used in addition to culture-based methods	266	65.3 (30–90)	2:1	Diabetes (36)	NR	Community-acquired pneumonia	*S. pneumoniae* 48 (18)
Alborzi et al., 2009 [41]	2006–2007	Iranian	Pilgrims with symptoms of ARI at Shiraz airport on return from the Hajj	Direct fluorescent staining and viral culture were performed on nasal wash specimens. Rhinovirus and enterovirus tested by RT-PCR	255	52.4 (19–82)	1:1.1	NR	Influenza vaccine (85.5)	Viral respiratory infections	Influenza 25 (9.8) Rhinovirus 15 (5.9) Other viruses 42 (16.4)
Alsayed et al., 2021 [14]	2019	Asian (64) African (30.3) European (2.7) North American (2.2) Oceanian (0.5)	Pilgrims with severe ARI symptoms who were initially hospitalised at seven healthcare facilities in Makkah, Arafat, and Mina at Hajj	Nasopharyngeal swabs were collected and tested using multiplex RT-PCR	185	58 (2 d–88 y)	1.9:1	NR	NR	Viral respiratory infections	Rhinovirus 53 (42) Influenza 54 (29.2) Coronaviruses 35 (18.9) Other viruses 19 (10.2) MERS-CoV 0 (0)
Alzeer et al., 1998 [32]	1994	Middle Eastern (34) South-East Asian (31) Indian (23) African (11)	All pilgrims admitted with pneumonia to Al-Noor Specialist Hospital and King Abdulaziz Hospital, Makkah	Sputum samples or fibre optic bronchoscopy samples for microscopy and culture, blood chemistry	64	63	2.6:1	NR	NR	Pneumonia	Viruses: Influenza 3 (4.7) Parainfluenza 1 (1.6) Bacteria: *M. tuberculosis* 13 (20.3) *S. pneumoniae* 6 (9.4) *K. pneumoniae* 5 (7.8) *H. influenzae* 3 (4.7) *S. aureus* 2 (3.1) Other bacteria 11 (17.3)
Annan et al., 2015 [42]	2013	Ghanaian	Adult pilgrims at the Kotoka International Airport after Hajj	Nasopharyngeal specimens were tested by RT-PCR	839	52 (21–85)	1:1.2	NR	NR	Viral respiratory infections	Rhinovirus 141 (16.8) RSV 43 (5.1) Influenza viruses 11 (1.3) MERS-CoV 0 (0)
Asghar et al., 2011 [15]	2005	Indonesian (18.4) Saudi (17.1) Pakistani (11.8) Indian (9.2) Egyptian (6.6) Malaysian (5.3) Syrian (4) Others (27.6)	Hajj pilgrims with suspected pneumonia who were admitted to hospitals in Makkah	Sputum samples were tested by routine culture, acid fast bacilli examination, and culture	141	>94% of cases were >50 y	1.3:1	Diabetes (55)	NR	Pneumonia	*M. tuberculosis* 1 (0.7) *S. pneumoniae* 5 (3.5) *K. pneumoniae* 8 (5.7) *S. aureus* 7 (5) Others 70 (50)
Ashshi et al., 2014 [36]	2010	280 Indonesian 157 Algerian 188 Indian 101 Syrian 128 Ivorian 104 Sierra Leonean 113 Somalian 123 Nigerian 112 Turkish 89 Australian 99 American 97 British	Pilgrims entering Saudi Arabia via King Abdulaziz International Airport, Jeddah city	Throat swab analysed using RT-PCR	1600	1309 aged > 40 291 aged ≤ 40	1.6:1	NR	Influenza vaccine (93.4)	Influenza A	Influenza A 120 (7.5)
Baharoon et al., 2009 [16]	2004	Majority were South Asian	All pilgrims admitted with severe sepsis and septic shock among Hajj pilgrims in two major ICUs in Makkah: King Faisal and King Abdulaziz Hospitals	Clinical diagnosis of severe sepsis/septic shock. Confirmation of source of infection with X-ray and/or positive culture	42	65.4	1.8:1	Lung diseases (71) Hypertension/Cardiac (43) Diabetes (29) Kidney diseases (2)	NR	Bacterial sepsis	Community-acquired pneumonia 23 (54.8)
Bokhary et al., 2022 [17]	2018	Egyptian (22.3) Sudanese (14.9) Algerian (11.6) Moroccan (9.1) Libyan (2.6) Saudi (14.9) Indian (7.4) Pakistani (6.6)	Adult pilgrims who sought medical care for upper RTIs during Hajj	Oropharyngeal swabs were taken and tested by bacteriological culture method and automated VITEK 2 COMPACT system	121	45	8.3:1	NR	NR	Bacterial respiratory infections	*H. influenzae* 6 (4.9) *S. pneumoniae* 2 (1.6) *S. aureus* 1 (0.8) Others 1 (0.8)
El-Gamal et al., 1988 [19]	1987	Pakistani, Indian and Indonesian (50)	Suspected cases of meningitis were presented to the outpatient clinic at King Abdulaziz Hospital in Al Madinah city	CSF, blood and skin samples were examined. CSF samples were examined under microscope and by latex test	229	(30–70)	2.3:1	NR	NR	Meningococcal	Meningitis 188 (82) (177 serogroup A and 11 serogroup C)
El-Kafrawy et al., 2022 [20]	2019	15 Indonesian 9 Indian 4 Moroccan 3 Somalian and Sudanese (each) 2 Saudi, Bangladeshi, and Nigerian (each) 13 others	Pilgrims with RTIs who presented to the healthcare facilities at Hajj sites in Makkah	Nasopharyngeal swabs were collected and tested by multiplex RT-PCR	53	Majority (58%) >60 years old	NR	NR	NR	Human rhinoviruses	Rhinoviruses 19 (36.8)
El-Sheikh et al., 1998 [21]	1991–1992	30 different nationalities	Pilgrims who were referred to either Ajyad hospital or three dispensaries in Jeddah. Upon enrolment, all patients were diagnosed with upper or lower RTIs	Sputa were collected and examined microscopically. Throat swabs were collected for cell culture and virus identification by immunofluorescence	1156	(10–80)	NR	NR	NR	Viral and bacterial respiratory infections	Viruses: RSV 18 (2.4) Influenza 49 (6.4) Parainfluenza virus 45 (5.9) Adenovirus 36 (4.7) Bacteria: *H. influenzae* 42 (10.6) *K. pneumoniae* 31 (7.8) *S. pneumoniae* 27 (6.8) *S. pyogenes* 7 (2.4) *S. aureus* 11 (3.8)
Hashem et al., 2019 [22]	2014	Majority Indian and Indonesian	During the 5 days of Hajj, samples were collected from all pilgrims presenting with ARI symptoms and suspected of MERS-CoV infection at seven healthcare facilities in Makkah, Mina, and Arafat	Nasopharyngeal samples were collected and tested using RT-PCR and microarray	132	61.85 (26–95)	2:1	NR	NR	Viral respiratory infections	Influenza 39 (29.5) Rhinoviruses 17 (12.1) Coronaviruses 24 (18.2) Other viruses 18 (13.6) MERS-CoV 0 (0)
Harimurti et al., 2021 [54]	2015	Indonesian	A multi-site longitudinal study collected data before the departure from Indonesia, and immediately upon arrival	Nasopharyngeal samples were collected pre- and post-Hajj and tested by culturing onto blood agar	813	53.1	1:1	Lung disease (2) Cardiovascular disease (0.8) Liver disease (0.2) Diabetes (5.8)	Pneumococcal vaccine (2)	Pneumococcal infection	*S. pneumoniae* (8.2)
Kandeel et al., 2011 [43]	2009	Egyptian	Random selection of pilgrims who returned by ship to Port Tawfiq. Non-random selection of returning pilgrims at Cairo International airport	Throat swabs analysed by RT-PCR	551	(12–65)	1.3:1	NR	Influenza vaccination (98)	Viral respiratory infections	Influenza A/H3N2 6 (1)
Karima et al., 2003 [23]	2000	Majority Pakistani (18) Indian (15) Indonesian (12)	All suspected and microbiologically confirmed cases of meningococcal disease referred to public and private hospitals and health centres in Makkah	CSF examination: Gram stain smears, latex agglutination and culture	105	23% (61–70) 22% (41–50)	1.9:1	fourteen diabetes one ischemic heart disease one renal failure three hypertension	NR	Meningococcal	Meningitis A, B, W 67 (64)
Koul et al., 2018 [44]	2014–2015	Indian	Pilgrims were interviewed for respiratory symptoms and tested for respiratory viruses in Srinagar International Airport, Jammu and Kashmir, India	Nasopharyngeal and throat swabs tested by RT-PCR	300	60 (26–60)	1:1.1	NR	Influenza vaccine (72)	Viral respiratory infections	Influenza 33 (11) Coronaviruses 52 (17.3) Rhinovirus 20 (6) Other viruses 20 (6) MERS-CoV 0 (0)
Lingappa et al., 2003 [24]	2000	African (35) Asian (42) Southwest Asian (26) European (4) Middle Eastern (19)	Case data from Ministry of Health surveillance records, regional health directories, clinical laboratory records and inpatient charts from all hospitals in Makkah, Al Madinah and Jeddah	Blood and CSF culture, latex agglutination of CSF	253	40 (0.2–80)	1.1:1	NR	NR	Meningococcal	253 confirmed cases among approx. 1.7 million pilgrims (0.015). Serogroups W 93 (37), A 60 (24), B 4 (2), C 4 (2)
Mandourah et al., 2012 [25]	2009–2010	Over 40 nationalities	All patients admitted to 15 hospitals in Makkah and Al Madinah	Sputum culture and blood culture	452	64	1.8:1	Diabetes (32.5) Lung diseases (17.1)	NR	Pneumonia	*S. pneumoniae* 3 (0.7) *K. pneumoniae* 5 (1.1) *S. aureus* 3 (0.7) Other bacteria 21 (4.6)
Mandourah et al., 2012 [26]	2009	East Asian (29.1) South Asian (24.6) Arab (30.9) Black (12.7) White (2.7)	All pilgrims admitted to ICUs of four key hospitals in Makkah	Nasal-swab specimens were tested by RT-PCR	110	60.5	1.7:1	Cardiovascular disease (28.2) Lung disease (20.9) Liver disease (6.4) Diabetes (28.2) Hypertension (31.8)	NR	Viral and bacterial respiratory infections	Influenza A/H1N1 * 24 (21.8) Community- acquired pneumonia 21 (19.3) Tuberculosis 1 (0.9)
Memish et al., 2012 [37]	2009	Middle Eastern (63) Asian or African (37)	On arrival of pilgrims just before Hajj, and before departure after Hajj at King Abdulaziz International Airport, Jeddah	Nasopharyngeal and throat swabs tested by xTAG Respiratory Viral Panel FAST assay. Specimens positive for influenza A but negative for seasonal H1 and H3 were subjected to additional PCR amplification to detect pandemic H1 and avian H5	3550	49.4	1.4:1	NR	Influenza vaccine (53.3)	Viral respiratory infections	Influenza 11 (0.5) RSV 8 (0.3) Coronaviruses 10 (0.4) Entero-rhinovirus 351 (13) Other viruses 14 (0.7)
Memish et al., 2014 [38]	2013	Majority from Indonesia (32.4) Pakistan (18.9) India (10.8)	All pilgrims admitted to 15 healthcare facilities in Makkah and Al Madinah, who were diagnosed with pneumonia	Sputum samples screened for MERS-CoV by RT-PCR and respiratory multiplex array was used to detect up to 22 other viral and bacterial respiratory pathogens	38	58.6 (25–83)	2.2:1	NR	NR	Viral and Bacterial respiratory infections	Viruses: Influenza 6 (15.8) Rhinoviruses 15 (39.5) Coronaviruses 5 (13.3) Others 2 (5.2) MERS-CoV 0 (0) Bacteria: *S. pneumoniae* 13 (34.2) *H. influenzae* 5 (13.2) Others 10 (26.3)
Memish et al., 2014 [39]	2013	Majority Indian (17.1) Indonesian (12.9) Pakistani (11.9) Turkish (10.7)	A random selection at the Hajj terminal of King Abdulaziz International Airport, Jeddah, of arriving and departing pilgrims	Nasal swabs analysed by RT-PCR	5235	51.8 (18–93)	1.2:1	Diabetes (6.8), Hypertension (13.1)	Influenza vaccine (22), pneumococcal vaccine (4.4)	MERS-CoV	MERS-CoV 0 (0)
Memish et al., 2015 [66]	2013	African (44.2) Asian (40.2) American (8.4) European (7.2)	Pilgrims recruited upon entering Saudi Arabia at Jeddah International Airport and at Mina camps before leaving Saudi Arabia	Nasal swabs analysed by RT-PCR	1206	50 (18–88)	1.9:1	NR	Influenza vaccine (21.9) Pneumococcal vaccine (1.2)	Viral and Bacterial respiratory infections	Viruses: Influenza (3.6) Rhinovirus (34.4) Coronaviruses (19.5) Others (2.4) Bacteria: Meningococcal (0.3) *S. pneumoniae* (12.7) *K. pneumoniae* (3.9) *S. aureus* (7.9) *H. influenzae* (11.7)
Moattari et al., 2012 [46]	2009	Iranian	Symptomatic Iranian pilgrims who arrived at Shiraz Airport Iran from Hajj	Throat swabs were collected and tested by virus culture and RT-PCR	275	46 (20–70)	1:1	NR	Influenza vaccine (100)	Influenza viruses	Influenza A/H3N2 8 (2.9) Influenza A/H1N1 5 (1.8) Influenza B 20 (7.3)
Muraduzzaman et al., 2018 [47]	2013–2016	Bangladeshi	Pilgrims and other people returning from the Middle East presented with respiratory symptoms were recruited via active screening at the point of entry	Nasal and throat swabs and sputum tested by RT-PCR	81	49 (14 m–81 y)	1.2:1	NR	NR	Viral respiratory infections	Influenza viruses 18 (22.2) Other viruses 6 (7.4) MERS-CoV 0 (0)
Refaey et al., 2017 [51]	2012–2015	Egyptian	A convenience sample of about 10% of pilgrims from each flight who returned at Cairo International Airport from Hajj	Nasopharyngeal swabs were collected from all participants while sputum specimens collected only from those with respiratory symptoms. Specimens were tested by RT-PCR	3364	56 (0–105)	(1:1.1)	NR	Influenza vaccine (19.7)	Viral respiratory infections	All influenza viruses 484 (14.4) Influenza A/H1N1 117 (3.5) Influenza A/H3N2 187 (5.6) Influenza B 180 (5.4) MERS-CoV 0 (0)
Shirah et al., 2017 [30]	2004–2013	African (43.9) Asian (51.2) European (3.3) North African (0.7) South American (0.47) Oceanian (0.47)	Clinical data of pilgrims with confirmed pneumonia who attended emergency department of a general hospital in Al Madinah during Hajj	Chest X-ray, throat and nose swabs, sputum and blood culture	1059	56.8 (48–64)	2.9:1	Lung disease (15) Diabetes (34.8) Cardiovascular disease (23.3)	NR	Pneumonia	*K. pneumoniae* 307 (29) *S. pneumoniae* 1 (1) *S. aureus* 382 (36) *H. influenzae* 257 (24) Other bacteria 102 (10)
Yavarian et al., 2018 [52]	2013–2016	Iranian	Specimens were taken from arriving pilgrims at Emam Khomeini Airport in Tehran	Throat swabs were collected and tested by using one-step RT-PCR	3840	NR	NR	NR	NR	Influenza viruses	All influenza viruses 499 (13) Influenza A/H1N1 258 (51.7) Influenza A/H3N2 100 (20) Influenza B 135 (27) MERS-CoV 0 (0)
Yezli et al., 2017 [35]	2015	Afghan (27.1) Pakistani (25.9) Bangladeshi (19.1) Nigerian (15.1) South African (12.7)	Non-hospitalised adult pilgrims were enrolled during Hajj who had coughs and could voluntarily produce sputum samples	Sputum samples were processed using the Xpert MTB-RIF assay	1164	54.5 (18–94)	2.6:1	Hypertension (58.8) Diabetes (42.2) Kidney disease (2) Lung disease (4.4) Liver disease (2.4) Cardiovascular disease (15.9)	NR	Tuberculosis	*M. tuberculosis* 15 (1.4)
Yousuf et al., 2000 [40]	1992–1993	Pakistani (53.3) Algerian (13.3) Indonesian, Thai, Syrian, American, and Congolese (6.7)	All patients admitted to King Abdulaziz Hospital, Al Madinah with a diagnosis of meningococcal disease	CSF culture and smear	15	NR	2.7:1	NR	NR	Meningococcal	Meningococcal infection A and C 8 (53.3)
Ziyaeyan et al., 2012 [53]	2009	Iranian	One in ten individuals passing through the passport checkout at Shiraz International Airport was selected	Pharyngeal swabs were collected and tested by using RT-PCR	305	49.2 (24–65)	1:1.3	NR	Influenza vaccine (97.7)	Influenza viruses	Influenza A/H1N1 4 (1.6) Other influenza A viruses 8 (2.6)

ARI: Acute respiratory infection; CSF: cerebrospinal fluid; ICU: intensive care unit; LMIC: low and middle-income countries; MERS-CoV: Middle East respiratory syndrome coronavirus; NR: not reported; PCR: polymerase chain reaction; RSV: respiratory syncytial virus; RTI: respiratory infection. * Likely influenza A (H1N1)pdm09.

**Table 2:** Summary of included studies involving participants from high-income countries.

Author, Publication Year	Study Year	Participants’ Nationalities (%)	Selection Method/Case Ascertainment	Testing Method	Sample Size	Mean/Median Age (Range)	Male: Female	Risk Factors (%)	Vaccine Uptake (%)	Detected Diseases Name	Disease Burden n (%)
Aberle et al., 2015 [55]	2014	Austrian	Returning Hajj pilgrims who had sought medical care in different Austrian hospitals/medical centres	Sera, sputa, throat swabs, or bronchoalveolar lavage samples were collected and tested by RT-PCR	7	54 (47–66)	(2.5:1)	Diabetes (43) Hypertension (43) Cardiovascular diseases (12.5) Lung disease (12.5)	NR	Viral respiratory infections	Influenza B 3 (43) Influenza A 2 (29) Rhinovirus 2 (29) MERS-CoV 0 (0)
Aguilera et al., 2002 [56]	2000	French and British	Hospitalised cases identified by National Surveillance Centres in Europe: only cases in France and UK were used for analysis	Cases were diagnosed by soluble antigen detection or PCR	90	51	(1:1.1)	NR	NR	Meningococcal	90 cases of meningococcal W135 disease: pilgrims 12 (13)
Al-Abdallat et al., 2017 [57]	2014	Jordanian	Returning Hajj pilgrims with symptoms of RTIs were instructed to present to sentinel health facilities in the south, north, and central regions of Jordan	Nasopharyngeal and oropharyngeal swabs were collected and tested by RT-PCR	125	51.5 (25–86)	(1.6:1)	Hypertension (22) Diabetes (14) Cardiovascular disease (8) Kidney disease (1) Lung disease (2)	NR	Viral respiratory infections	Rhino/enterovirus 59 (47) Coronavirus 16 (13) Influenza 6 (5) Other viruses 5 (4) MERS-CoV 0 (0)
Alahmari et al., 2022 [67]	2021	100 different nationalities	Records of pilgrims who did the PCR test were collected from the official database of the Saudi Ministry of Health	PCR-based surveillance with paired-swab samples (pre-Hajj and post-Hajj)	58428	NR	(1:1)	NR	NR	COVID-19 infection	SARS-CoV-2 41 (0.1)
Alfelali et al., 2020 [31]	2013–2015	Saudi and Qatari (91) Australian (9)	Pilgrims were randomised to ‘facemask’ or ‘no facemask’ by tents in Mina, Makkah	Nasal swabs were collected and tested using a multiplex RT-PCR	7687	34 (18–95)	(1:1.1)	NR	Influenza vaccine: intervention group (49.9) vs. control group (49.4)	Viral respiratory infections	Rhinovirus (35.1) Influenza A/H1N1 and H3N2 (4.5) Parainfluenza (1.7)
Alzeer et al., 2023 [70]	2018	NR	Samples were collected voluntarily from pilgrims who resided in the study tents	Nasopharyngeal swabs were collected and tested by multiplex RT-PCR	32	NR	NR	NR	NR	Viral and bacterial respiratory infections	Viruses: Rhinovirus 5 (15.62) Coronavirus 3 (9.4) Influenza 3 (9.4) Other viruses 7 (21.9) Bacteria: *K. pneumoniae* 20 (62.5) *S. aureus* 10 (31.3) *S. pneumoniae* 5 (15.6) *H. influenzae* 1 (3.1) Other bacteria 3 (9.4)
Atabani et al., 2016 [58]	2013–2015	British	UK travellers/pilgrims who returned from the Middle East and presented to hospitals in the Midlands, the Southwest, and North of England with RTI symptoms were actively investigated	Nose and throat swabs, nasopharyngeal aspirates, sputum and bronchoalveolar lavage samples were collected and tested by RT-PCR	202	54 (4 m–85 y)	(1.4:1)	NR	NR	Viral respiratory infections	Influenza 41 (20.3) Rhinovirus 29 (14.4) Other viruses 20 (9.9) MERS-CoV 0 (0)
Balkhy et al., 2004 [18]	2003	Saudi (46.8)	Patients presented to the National Guard Mina hospital outpatient clinic, on days 10 and 11 of Hajj	Throat swabs were inoculated onto MDCK, A549 and LL19Ks cell lines using conventional methodology, and screened by immunofluorescence for viruses	500	Majority (20–40)	(1.1:1)	NR	Influenza vaccine (4.4)	Viral respiratory infections	Influenza 30 (7) Other viruses 24 (4.8)
Barasheed et al., 2014 [33]	2013	Australian, Saudi, and Qatari	Pilgrims were recruited during the first day of Hajj and followed closely for four days	Nasopharyngeal swabs were collected and tested by multiplex RT-PCR	112	35 (18–75)	(1:1.3)	Lung disease (68) Diabetes (41) Cardiovascular disease (4) Kidney disease (4)	Influenza vaccine (68.8)	Viral respiratory infections	Rhinovirus 28 (25) Influenza 5 (4) Coronavirus 2 (2) Other viruses 5 (4)
Barasheed et al., 2014 [34]	2011	Australian	Tents were randomised to ‘supervised mask use’ versus ‘no supervised mask use’. Pilgrims with ILI symptoms for ≤3 days were recruited as ‘cases’ and those who slept within 2 m of them as ‘contacts’	Nasal swabs were taken and tested using Quick-Vue A+B point-of-care test and NAT for influenza viruses	164	44.1 (17–80)	(1:1.3)	NR	NR	Viral respiratory infections	Rhinovirus 39 (23.8) Influenza 7 (4.3) Other viruses 2 (1.2)
Benkouiten et al., 2013 [60]	2012	French	A prospective survey among a cohort of pilgrims departing from Marseille, France, to Makkah for Hajj	Nasal swabs were collected and tested by RT-PCR	154	59.3 (21–83)	(1:1.6)	Diabetes (27.5) Hypertension (26.3) Lung disease (7.8) Cardiovascular disease (7.2)	Influenza vaccine (45.6) Pneumococcal vaccine (35.9)	Viral respiratory infections	Rhinovirus 13 (8.4) Influenza 2 (1.3) Other viruses 4 (2.6)
Benkouiten et al., 2014 [61]	2013	French	Pilgrims were recruited at a private specialised travel agency in Marseille, France. participants were sampled and followed up before departing from France and before leaving Saudi Arabia	Paired nasal and throat swab specimens were collected and tested by RT-PCR	129	61.7 (34–85)	(1:1.5)	NR	Influenza vaccine (0) Pneumococcal vaccine (51.2)	Viral and bacterial respiratory infections	Viruses: Influenza 10 (7.8) Coronavirus 27 (20.9) Rhinovirus 19 (14.7) MERS-CoV 0(0) Bacteria: *S. pneumoniae* 80 (62)
El Bashir et al., 2004 [65]	2003	British	A cohort of pilgrims from the East End of London who participated in Hajj	Blood samples were collected pre-and post-Hajj, and tested by haemagglutination inhibition	115	NR	NR	NR	Influenza vaccine (26)	Influenza viruses	All influenza viruses 44 (38) Influenza A/H3N2 42 (37) Influenza A/H1N1 1 (0.9) Influenza B 1 (0.9)
Erdem et al., 2016 [59]	201–2015	Turkish	In patients with a diagnosis of a travel-associated infection needing hospitalisation after returning from the Arabian Peninsula. Data were collected retrospectively from infectious diseases departments of 15 Turkish referral centres	Microbiological cultures were used for bacteria and Multiplex/RT-PCR was used for viruses	185	60.3	(1:1.1)	Diabetes (24.3) Hypertension (8.6) Cardiovascular disease (10.8) Liver disease (1.1) Kidney disease (0.54) Lung disease (14.1)	NR	Viral and bacterial respiratory infections	Viruses: Influenza 15 (8.1) Coronavirus 1 (0.5) Rhinovirus 1 (0.5) Other viruses 3 (1.6) Bacteria: *S. pneumoniae* 1 (0.5) *H. influenzae* 1 (0.5) Other bacteria 1 (0.5)
Hoang et al., 2022 [62]	2014–2018	French	Hajj pilgrims from Marseille, France, were recruited through a private specialised travel agency and were systematically sampled before departing and upon their return from Hajj	Nasopharyngeal swabs were collected and tested by RT-PCR	207	NR	NR	Diabetes (32.4) Hypertension (28) Lung disease (15) Cardiovascular disease (9.7) Kidney disease (1.9)	Influenza vaccine (29.5) Pneumococcal vaccine (30.9)	Viral and bacterial respiratory infections	Viruses: Rhinovirus (40.6) Coronavirus (15.5) Influenza (2.9) Bacteria: *S. aureus* (35.8) *H. influenzae* (30.4) *K. pneumoniae* (17.4) *S. pneumoniae* (3.9)
Jones et al., 1990 [68]	1987–1988	British	Meningococcal Reference Laboratory	Nasal swabs were cultured	39	NR	NR	NR	NR	Meningococcal	Meningococcal A (0.18)
Ma et al., 2017 [45]	2013–2015	Chinese	Randomly selected returning pilgrims arriving at Xinjiang and Gansu airports	Viral infection samples collected and tested by RT-PCR	847	2.24	(1.4:1)	NR	Influenza vaccine (100)	Viral respiratory infections	Influenza 48 (5.7) Coronavirus 3 (0.3) MERS-CoV 0 (0)
Marglani et al., 2016 [27]	2014	Gulf (58) Asian (12.4) South Asian (11.9) North African (11.5) African (3.5) European (2.2) American (0.5)	Patients presented to the emergency or outpatient departments of Alnoor Specialised Hospital in Makkah	Bacteriological culture and isolation, and antimicrobial susceptibility testing (AST) were performed using MicroScan Walk Away System ID/AST	226	34.6 (9–77)	(3.5:1)	NR	NR	Bacterial acute rhinosinusitis	*S. aureus* 46 (20.3)
Matsika-Claquin et al., 2001 [69]	2000	French	Standardised questionnaire used to interrogate study subjects	A case was considered to be confirmed when the strain isolated from usually sterile media was found to be identical to the epidemic strain (W135, 2a: P1-2.5--clonal complex ET37)	27	(2 m–87 y)	(1:1.2)	NR	NR	Meningococcal	Meningococcal W (0.002)
Nik Zuraina et al., 2018 [48]	2016	Malaysian	Hajj pilgrims who returned to the arrival hall of Sultan Ismail Airport, Kota Bharu, Kelantan, Malaysia with RTI symptoms, including productive cough	Bacteriological culture method and Vitek II system	297	57.4 (27–82)	(1:1)	NR	NR	Bacterial respiratory infections	*H. influenzae* 123 (44.4) *K. pneumoniae* 11 (3.7) *S. pneumoniae* 2 (0.7) Other bacteria 28 (9.4)
Nik Zuraina et al., 2022 [49]	2016	Malaysian	Hajj pilgrims who returned to Sultan Ismail Petra Airport, Kelantan, Malaysia with RTI symptoms during Hajj	Sputum specimens were collected and tested by culture and thermostabilized, multiplex PCR	202	56.7 (26–80)	(1:1.1)	NR	NR	Bacterial respiratory infections	*H. influenzae* 88 (43.5) *K. pneumoniae* 1 (0.5) *S. pneumoniae* 2 (1) Other bacteria 20 (9.9)
Novelli et al., 1987 [50]	1987	Qatari	Returning pilgrims or their contacts admitted to Hamad General Hospital, Doha, Qatar	Blood and/or CSF culture and/or latex test for meningococcal antigen in CSF	15	40	NR	NR	NR	Meningococcal	Meningococcal A 6 (40)
Rashid et al., 2008 [28]	2005	British	Pilgrims attending the British Hajj Delegation Medical Clinic in Makkah and in tents in Mina with symptoms of upper RTI	Two nasal swabs taken and tested by testing using PoCT and RT-PCR	202	44 (1.5–83)	(9.1:1)	Diabetes, chronic heart, lung or kidney disease, all high-risk conditions together (26)	Influenza vaccine (28)	Viral respiratory infections	Influenza 28 (13.9) RSV 9 (4)
Rashid et al., 2008 [29]	2006	British	Hajj pilgrims with upper RTI who attended British Hajj Delegation Clinic in Makkah and Mina	Two nasal swabs taken and tested by testing using PoCT and RT-PCR	150	41 (14–81)	(11.5:1)	Diabetes (62) Lung disease (23) Cardiovascular disease (12)	Influenza vaccine (37.3)	Viral respiratory infections	Influenza 17 (11) Rhinovirus 19 (12.7) Other viruses 2 (1.3)
Wilder-Smith et al., 2003 [63]	2002	Singaporean	Referred by travel agencies, pilgrims were recruited at a Muslim centre in Singapore that performs mass vaccinations and following a few months after Hajj	Paired blood samples were collected pre- and post-Hajj, and IgG antibodies for pertussis whole-cell antigen were measured	358	48 (16–75)	(1:1.7)	NR	NR	Pertussis	*Bordetella pertussis* 5 (1.4)
Wilder-Smith et al., 2005 [64]	2002	Singaporean	Pilgrims were consecutively recruited at mass pre-travel vaccination sites before Hajj and following a few months after Hajj	A whole-blood assay (Quanti- FERON TB assay) prior to departure and 3 months after return from Hajj	365	49.2 (18–75)	(1:1.6)	Diabetes (8.8)	NR	Tuberculosis	*M. tuberculosis* 139 (38)

CSF: cerebrospinal fluid; MERS-CoV: Middle East respiratory syndrome coronavirus; NR: not reported; PCR: polymerase chain reaction; RTI: respiratory tract infection; RSV: respiratory syncytial virus; UK: United Kingdom. PoCT: point-of-care testing.

### 3.2. Comparative Variables and Attack Rates of Hajj-Acquired Airborne Infections

Table 3 provides a detailed comparison of the demographic data, risk factors, vaccination uptake, and burden of confirmed airborne infections between pilgrims from LMIC and HIC. In the pooled sample, there were 27,799 pilgrims from LMIC with ages ranging from 2 days to 105 years and a male-to-female ratio of 1.3:1, and 70,865 pilgrims from HIC with ages between 2 months and 95 years and an equal gender ratio of 1:1. Most pilgrims from LMIC arrived from Africa, Southeast Asia, and the Eastern Mediterranean, whereas most pilgrims from HIC mainly arrived from Europe, the Eastern Mediterranean, and the Western Pacific.

The most commonly reported chronic medical conditions among pilgrims from LMIC were chronic lung and cardiovascular diseases; noting that, the highest proportions of these conditions (71% and 43%, respectively) were observed in one study that studied a highly selective cohort of pilgrims admitted to ICU with severe sepsis and septic shock [16]. On the other hand, the most frequently observed chronic medical conditions among pilgrims arriving from HIC were variable, with diabetes ranging from 1% among pilgrims from Australia [34] to 32% among pilgrims from France [62], and hypertension ranging from 9% among pilgrims from Turkey [59] to 28% among pilgrims from France [62].

Studies that reported the pilgrims’ vaccination uptake revealed a wide variation in uptake both among LMIC and HIC pilgrims. Influenza vaccine uptake rates in LMIC ranged from 20% among Egyptian Hajj pilgrims in the years 2012 to 2015 [51] to 100% among Iranian pilgrims in 2009 [46]. As for HIC, a study involving French pilgrims in 2013 found that none of them had received an influenza vaccine before departing for Hajj, due to the unavailability of the vaccine at that time [60], while another study involving Chinese pilgrims indicated that all of them had received the vaccine in the years 2013 and 2015 [45]. The uptake rates of the pneumococcal vaccine in LMIC varied between 1% and 4% among Hajj pilgrims during the year 2013 [38,39]. Regarding HIC, a study conducted among French pilgrims between 2014 and 2018 revealed that 31% of them received the vaccine before embarking on their Hajj journey [62]; in contrast, a separate study conducted on French pilgrims in 2013 reported that 51% of them had received the vaccine [60].

Epidemiological patterns of proven viral airborne infections seemed to show higher attack rates of viral infections among HIC pilgrims than among LMIC pilgrims. Human rhinoviruses were the most prevalent viral agent among pilgrims in both groups, followed by influenza and human coronaviruses, including SARS-CoV-2. On the other hand, the attack rate of confirmed bacterial airborne infections varied more widely across the groups. For instance, pilgrims from LMIC tended to have higher rates of meningococcal and *Staphylococcus aureus* infections, whereas pilgrims from HIC seemed to have higher rates of other bacterial infections such as *M. tuberculosis*, *Streptococcus pneumoniae, Klebsiella pneumoniae*, and *Haemophilus influenza* infections (see Table 3 for detailed comparative results).

Multiple studies have investigated the emerging viral airborne infections during Hajj over the past few years. For instance, five studies investigated influenza A (H1N1)pdm09: Ashshi et al. [36] reported nine (0.6%) confirmed cases; Kandeel et al. [43] reported not detecting any case; Memish et al. [37] reported two (0.1%) cases; Moattari et al. [46] recorded five (1.8%) cases; and Ziyaeyan et al. [53] reported another five (1.6%) cases. Furthermore, 12 studies were conducted between 2013 and 2015 to identify the occurrence of MERS-CoV during the pilgrimage; nonetheless, no case of MERS-CoV virus was confirmed [22,38,42,44,45,47,51,52,55,57,58,60]. Recently, only one retrospective surveillance study assessed the rate of COVID-19 among Hajj pilgrims amidst the pandemic in 2021, which found that 41 pilgrims out of a total of 58,428 tested positive for COVID-19 during and after Hajj [67].

In terms of quality assessment of the included studies, most LMIC and HIC studies were average in quality, as defined by a NOS score of total stars of between three and five; however, four LMIC studies [37,38,39,54] and nine HIC studies [31,33,34,56,60,61,62,65,67] received a good quality rating of between six and nine stars (Table 4).

## 4. Discussion

This systematised review presented the epidemiological patterns of airborne infections acquired during Hajj among pilgrims from LMIC and HIC. The qualitatively synthesised data showed that pilgrims from both resource-rich and resource-poor settings are at risk of acquiring airborne diseases during Hajj, but some infections are more common in LMIC or HIC than others, and vice versa. Hajj is an overcrowded event, and the proximity of participants, along with environmental conditions, amplifies the risk of transmitting respiratory pathogens, thus contributing to outbreaks of airborne infections [2]. Previously published systematic reviews also reported the occurrence of airborne/respiratory infections to be common among pilgrims. For instance, in a review of studies published prior to February 2018 reporting the prevalence of symptomatic respiratory infections among Hajj pilgrims, Benkouiten et al. found that influenza-like illness (ILI) ranged from 8% to 78% and pneumonia from 0.2% to 55% [9]. Safarpour et al. demonstrated in a meta-analysis that all serotypes of influenza viruses were identified among pilgrims, with estimated prevalence rates of 3.6%, 2.9%, and 0.9% for influenza types A, B, and C, respectively [10].

This systematised review uniquely shows wide variations in the burden of airborne infections between LMIC and HIC pilgrims. Despite the fact that Hajj pilgrims hail from resource-rich salubrious countries and therefore have better compliance with, and awareness about, the preventive measures, and access to more advanced public health services, the microbiological data paradoxically show that they may be at a higher (or at least at an equal) risk of developing viral infections compared to those from LMIC. On the other hand, the attack rates of bacterial infections demonstrated a more varied pattern. Pilgrims from LMIC seemed to have higher rates of certain bacterial infections, such as meningococcal infection, as was found among 82% of pilgrims with suspected infection at the time of an outbreak during the 1987 Hajj season [19], whereas some other bacterial infections were more prevalent among HIC pilgrims, for instance, *M. tuberculosis* infection, as was recorded among 38% of Singaporean Hajj pilgrims after the year 2002 pilgrimage [64].

This wide range of yielded rates may have been attributed to disparities in study settings, sample types, pilgrims’ pre-existing immunity including previous infection, and diagnostic methods used. A number of other factors may have also contributed to these differences. For instance, pilgrims participating in Hajj either from developed or developing countries share equally the same rituals as instructed by Islam; there is no discrimination between pilgrims based on their gender, income, or ethnicity, so HIC pilgrims were not actually at an advantage compared to their LMIC counterparts. Furthermore, differences in hygiene practices, living conditions, and sociodemographic factors may have influenced the transmission dynamics of such infections among pilgrims. Due to disparities in healthcare expenses, infrastructure, and resources, including diagnostic capacity, there could be variations in diagnostic yields for infectious pathogens between HIC and LMIC. For instance, HIC may employ more sophisticated and expensive diagnostic protocols, leading to more accurate and comprehensive data, while LMIC may rely on less-advanced diagnostic methods, potentially resulting in variations in the reported incidence of infections.

Although vaccination against influenza is strongly encouraged as part of pre-Hajj health preparations for all pilgrims, the results showed a wide variability in vaccination uptake, likely due to differences in seasonal vaccine availability and vaccination policies across countries. This was clearly noticed among pilgrims from France during the 2013 Hajj season, where none of them managed to receive an influenza vaccine before departing for Hajj, because they needed to set out on Hajj before the seasonal vaccine was available in their jurisdictions [60]. The Scientific Committee for Influenza and Pneumococcal Vaccination guidelines as part of the Saudi Thoracic Society recommends the following for Hajj pilgrims: all persons of ≥50 years are recommended to receive a combined vaccination with a 23-valent pneumococcal polysaccharide vaccine (PPSV23) and a 13-valent pneumococcal conjugate vaccine (PCV13) before Hajj (for those planning immediately before Hajj, at least one dose of PPSV23), immunocompetent persons of <50 years with risk factors are recommended to receive a single dose of PPSV23 at least 3 weeks before Hajj, and it is not recommended that the vaccine be routinely provided to healthy persons aged <50 years [71]. However, the uptake of a pneumococcal vaccine is suboptimal among the general population of KSA and other Gulf countries. For example, only 6% of Saudi adults have received the vaccination amidst the COVID-19 pandemic [72]. It is common for HIC to implement a policy that suggests or provides pneumococcal vaccination for elderly individuals and those with pre-existing medical conditions [73]; consequently, a significant number of pilgrims from these countries would likely have received the pneumococcal vaccine [5].

These findings emphasise the need for targeted interventions, such as enhanced pre-Hajj vaccination and awareness programs for all pilgrims, regardless of their income status. Non-pharmaceutical interventions (e.g., facemasks, hand hygiene, cough etiquette, and physical distancing) have been used to prevent airborne infections at Hajj, but these interventions are underutilised, or at least the evidence of their effectiveness among Hajj pilgrims is inconclusive or unproven. For instance, a large-scale randomised controlled trial (RCT) among pilgrims found that the efficacy of using a facemask against viral respiratory infections was inconclusive [31]. Similarly, a recent pilot RCT conducted during the COVID-19 pandemic among Umrah participants (the abbreviated pilgrimage that Muslims can take year-round) failed to yield conclusive evidence regarding the protective effects of hand hygiene [74].

As the host country of this MG event, KSA has demonstrated notable success in safeguarding the well-being and safety of participants in the modern era through advance planning, comprehensive healthcare plans, preventive strategies, and risk-based infection control measures to contain the health risks associated with this pilgrimage [75]. The significant contribution of KSA in preventing and controlling the dissemination of emerging infectious diseases in recent Hajj seasons has been lauded worldwide and proven by some of the studies included in this review. The low prevalence of pandemic infections like COVID-19 and influenza A (H1N1)pdm09, and the absence of MERS-CoV at Hajj are testament to this.

A key limitation of this review is limiting the search strategy to only English language articles, but because most original Hajj-related manuscripts are generally published in English, the possibility of missing any important publication is very remote. An additional limitation was the clinical heterogeneity of the included studies, meaning that a meta-analysis was not feasible. Also, the way the results were presented in the included papers precluded us from carrying out any quantitative synthesis. We presented the results as lower and upper ranges of proportions and rates. We have called this a ‘systematised review’ because not all standard steps of a ‘systematic review’ could be followed; for instance, this review was not registered in PROSPERO, although it utilised a rigorous and comprehensive study design, including adherence to the PRISMA statement and the use of the NOS quality assessment tool. Furthermore, databases other than PubMed were not searched because from our experience most good-quality Hajj-related manuscripts are indexed in PubMed. There could be some manuscripts that are published in non-PubMed-indexed journals. Finally, we could not focus on other parallel information about the identified pathogens including their genetic characteristics, virulence, or drug resistance. Despite these limitations, this study significantly contributed by shedding light on the epidemiological patterns of airborne infections among attendees of MG events, which hold substantial implications for policymakers in host countries and offer valuable information that can inform decision-making processes. Given the recent announcement by the World Health Organization (WHO) that COVID-19 no longer constitutes a public health emergency of international concern, coupled with the anticipated increase in the number of Hajj participants in 2023 [76,77], it becomes important to direct future studies to provide a head-to-head comparison of the epidemiological patterns of Hajj-acquired airborne infections among pilgrims from LMIC and HIC using similar (if not identical) study settings.

## 5. Conclusions

This review comprehensively assessed the burden and epidemiological patterns of airborne infections among Hajj pilgrims from LMIC and HIC. The findings showed that pilgrims from both regions are at risk of acquiring airborne diseases during Hajj. More Hajj pilgrims from HIC seemed to develop viral respiratory infections compared to those from LMIC; however, such a difference did not seem to exist for bacterial respiratory infections. The findings highlight the need for improved vaccination coverage and effective infection control measures to prevent the spread of airborne infections during the Hajj pilgrimage and ensure the safety and well-being of all pilgrims, irrespective of their socioeconomic status or country of origin.

## Figures and Tables

**Table 3:** Comparative variables and attack rates of airborne infections between low and middle-income and high-income countries.

Variables	Low and Middle-Income Countries	High-Income Countries
**Demographics**		
Pooled study sample	27,799	70,865
Age (range)	2 d–105 y	2 m–95 y
Male: Female	1.3:1	1:1
**Vaccination uptake (%)**		
Influenza vaccination	19.7–100	0–100
Pneumococcal vaccination	1.2–4.4	30.9–51.2
**Risk factors (%)**		
Diabetes	5.8–36	0.9–32.4
Hypertension	2.9–43	8.6–28
Cardiovascular disease	0.8–43	0.1–10.8
Chronic lung disease	1.1–71	1.4–15
Chronic kidney disease	0.5–2	0.1–1.9
Chronic liver disease	0.2–6.4	1.1
**Attack rate of viral airborne infections (%)**		
Influenza	0.5–29.5	1.3–38
Human rhinovirus	5.9–39.5	0.5–47
Human coronavirus	0.4–19.5	0.1–20.9
Other viruses	0.7–16.4	0.7–21.9
**Attack rate of bacterial airborne infections (%)**		
Meningococci	0.015–82	0.002–40
*M. tuberculosis*	0.7–20.3	38
*S. pneumoniae*	1–54.8	0.5–62
*K. pneumoniae*	1.1–29	0.5–62.5
*H. influenza*	4.7–24	0.5–44.4
*S. aureus*	0.7–36	20.3–35.8
Other bacteria	0.8–50	0.5–9.9

**Table 4:** Newcastle and Ottawa Scale (NOS) scores for the included studies.

Studies	Selection (Max 4 Stars)	Comparability (Max 2 Stars)	Outcome/Exposure (Max 3 Stars)	Total Score
High-income countries (HIC)				
Aberle et al., 2015 [55]	**	–	**	4
Aguilera et al., 2002 [56]	***	–	***	6
Al-Abdallat et al., 2017 [57]	**	–	**	4
Alahmari et al., 2022 [67]	***	–	***	6
Alfelali et al., 2020 [31]	***	*	**	6
Alzeer et al., 2023 [70]	***	–	**	5
Atabani et al., 2016 [58]	**	–	**	4
Balkhy et al., 2004 [18]	**	–	**	4
Barasheed et al., 2014 [33]	***	*	**	6
Barasheed et al., 2014 [34]	***	*	**	6
Benkouiten et al., 2013 [60]	***	–	***	6
Benkouiten et al., 2014 [61]	***	–	***	6
El Bashir et al., 2004 [65]	***	–	***	6
Erdem et al., 2016 [59]	**	–	***	5
Hoang et al., 2022 [62]	***	–	***	6
Jones et al., 1990 [68]	**	–	**	4
Ma et al., 2017 [45]	**	–	***	5
Marglani et al., 2016 [27]	**	–	**	4
Matsika-Claquin et al., 2001 [69]	**	–	**	4
Nik Zuraina et al., 2018 [48]	**	–	**	4
Nik Zuraina et al., 2022 [49]	**	–	**	4
Novelli et al., 1987 [50]	**	–	**	4
Rashid et al., 2008 [28]	**	–	***	5
Rashid et al., 2008 [29]	**	–	***	5
Wilder-Smith et al., 2003 [63]	***	–	**	5
Wilder-Smith et al., 2005 [64]	***	–	**	5
Low and middle-income countries (LMIC)				
AlBarrak et al., 2018 [13]	**	–	**	4
Alborzi et al., 2009 [41]	**	–	**	4
Alsayed et al., 2021 [14]	**	–	**	4
Alzeer et al., 1998 [32]	**	–	**	4
Annan et al., 2015 [42]	**	–	**	4
Asghar et al., 2011 [15]	**	–	**	4
Ashshi et al., 2014 [36]	**	–	**	4
Baharoon et al., 2009 [16]	**	–	**	4
Bokhary et al., 2022 [17]	**	–	*	3
El-Gamal et al., 1988 [19]	**	–	**	4
El-Kafrawy et al., 2022 [20]	**	–	**	4
El-Sheikh et al., 1998 [21]	**	–	**	4
Hashem et al., 2019 [22]	**	–	**	4
Harimurti et al., 2021 [54]	***	–	***	6
Kandeel et al., 2011 [43]	**	–	**	4
Karima et al., 2003 [23]	**	–	**	4
Koul et al., 2018 [44]	**	–	**	4
Lingappa et al., 2003 [24]	**	–	**	4
Mandourah et al., 2012 [25]	**	–	**	4
Mandourah et al., 2012 [26]	**	–	**	4
Memish et al., 2012 [37]	***	–	***	6
Memish et al., 2014 [38]	**	–	**	4
Memish et al., 2014 [66]	***	–	***	6
Memish et al., 2015 [39]	***	–	***	6
Moattari et al., 2012 [46]	**	–	**	4
Muraduzzaman et al., 2018 [47]	**	–	**	4
Refaey et al., 2017 [51]	***	–	**	5
Shirah et al., 2017 [30]	**	–	**	4
Yavarian et al., 2018 [52]	**	–	**	4
Yezli et al., 2017 [35]	**	–	**	4
Yousuf et al., 2000 [40]	**	–	**	4
Ziyaeyan et al., 2012 [53]	**	–	**	4

Asterisks represent score points.

## Data Availability

Not applicable.
